# Communicating and understanding pain: Limitations of pain scales for patients with sickle cell disorder and other painful conditions

**DOI:** 10.1177/1359105320944987

**Published:** 2020-08-01

**Authors:** Peter J Collins, Alicia Renedo, Cicely A Marston

**Affiliations:** 1 University of Greenwich, UK; 2 Munich Center for Mathematical Philosophy, Ludwig-Maximilian-University, Munich, Germany; 3 London School of Hygiene and Tropical Medicine, UK

**Keywords:** context, pain, pain communication, pain management, pain scales, qualitative methods, sickle cell disease, sickle cell disorder, social interaction, trust

## Abstract

Pain communication in healthcare is challenging. We examine use of pain scales to communicate pain severity via a case study of people with sickle cell disorder (SCD). We show how pain communication involves complex social interactions between patients, healthcare professionals and significant others – none of which are included in pain ratings. Failure to account for relational aspects of pain may cause problems for any patient. For SCD, mutual distrust shapes pain communication, further complicating clinical assessments. Moreover, SCD pain is particularly severe, making ratings hard to interpret compared with ratings from non-SCD patients, potentially exacerbating problems in managing pain relief.

Managing pain can be extremely challenging for individuals and healthcare providers (Hadjistavropoulos et al., 2011). Pain – ‘an unpleasant sensory and emotional experience associated with actual or potential tissue damage, or described in terms of such damage’ (IASP, 2017 Paragraph 1) – is particularly challenging because of its nature; it is a private sensory experience and so must be inferred from observation or communicated. One form of communication – self-report – has become a ‘gold standard’ (Schiavenato and Craig, 2010). Self-report is ubiquitous and frequently facilitated with pain scales (Schiavenato and Craig, 2010), tools described as reliable and valid (Broderick et al., 2006; Clark et al., 2003; Ferreira-Valente et al., 2011).

While pain has been treated as a fifth vital sign (American Pain Society, 1999) – an objective indication of how well the body is currently functioning – it is subject to various social and contextual influences (Hadjistavropoulos et al., 2011), including, crucially, trust between healthcare provider and patient (Schiavenato and Craig, 2010).

A useful case to examine pain communication is sickle cell disorder (SCD), an inherited blood disorder characterised by chronic and acute painful episodes (National Institute for Health and Clinical Excellence (UK) [NICE], 2012); chronic organ damage; and reduced life expectancy (Chakravorty et al., 2018; Piel et al., 2017). Considering the example of SCD is valuable to understand pain communication for three key reasons: First, SCD exemplifies issues of trust; second, it is characterised by severe pain episodes; and third, there is an urgent need for better pain management in SCD.

Trust (or lack of trust) is particularly important in SCD (Chakravorty et al., 2018; Dyson et al., 2010). SCD patients’ pain reports are not always taken seriously by providers (Miles et al., 2019; Mulchan et al., 2016; Renedo et al., 2019), perhaps partly because SCD patients do not necessarily have any signs of pain visible to the provider. Self-reports of pain may also be disregarded because of stigma and stereotyping of SCD patients related to their need for opioid-based analgesics (Maxwell et al., 1999), with patients sometimes mischaracterised as ‘drug-seekers’ (Haywood et al., 2009; Labbé et al., 2005; Maxwell et al., 1999; Renedo et al., 2019; Shapiro et al., 1997), that is, as wanting medication for reasons other than pain relief, such as opioid addiction (McCaffery et al., 2005). SCD is a racialised condition (Bediako and Moffitt, 2011), further increasing the potential for distrust, as we will see in later sections.

SCD is characterised by episodic acute pain, with many patients also reporting chronic pain (Adegbola et al., 2012; Dampier et al., 2017). SCD pain is highly complex, and severe SCD pain ‘waxes and wanes, relapses, and remits in a recurrent and unpredictable fashion’ (Ballas et al., 2012, p. 3653). Such qualities may push pain scales to their limits and lessons from the case of SCD – a condition where pain is so dominant and so complex in quality – may be particularly helpful to inform work on pain management for other conditions.

Numerous studies have indicated that management of SCD pain can be poor, particularly for patients in emergency departments during painful episodes (Chakravorty et al., 2018; Renedo et al., 2019; Tanabe et al., 2007, 2010). Improvements are urgently needed (Chakravorty et al., 2018; Renedo et al., 2019). Delays are more common for patients with SCD than other conditions (Lazio et al., 2010) and such delays lead to avoidable suffering and morbidity (Wilson and Nelson, 2015). Expectations of poor clinical care in turn affect SCD patient pain expression and care seeking during painful episodes (Jenerette and Brewer, 2010; Renedo et al., 2019).

## Literature review

### Types of scales and their limitations

Healthcare providers use various pain scales: for instance, rank-ordered descriptors, a line with verbal anchors, picture or face scales, and numerical scales (for discussion, see Ferreira-Valente et al., 2011). Scales feature in clinical guidelines, helping to identify treatments. Current UK guidelines state that providers should use age-appropriate scoring tools when assessing painful episodes in SCD, classifying ratings of 4 to 7 on the Visual Analogue Scale, or an equivalent scale, as moderate pain and ratings above 7 as severe (NICE, 2012). These classifications factor into provider decision making about pain relief (NICE, 2012). This use of scales implies that (some) users believe that scales can provide objective measurements, or at the very least provide a meaningful measure to inform clinical responses.

Experimental studies in laboratory settings support scales’ reliability and validity (Broderick et al., 2006; Clark et al., 2003; Ferreira-Valente et al., 2011) but neglect social and contextual influences outside the study environments. For example, validation studies manipulate pain’s sensory dimension, but do not account for the ways in which real-world pain experience is multi-dimensional (Hadjistavropoulos et al., 2011). For instance, pain has cognitive dimensions, with catastrophizing – including rumination and magnification – being associated with poor treatment outcomes (Hadjistavropoulos et al., 2011). It also has affective dimensions, with the sensory experience being moderated by distress, confusion and anxiety (Hadjistavropoulos et al., 2011). Such dimensions cannot be differentiated using many standardised scales (Craig, 2009; Schiavenato and Craig, 2010; Tait and Chibnall, 2014). In the case of SCD pain, pain experience is complex both at the physical and interpersonal (provider-patient) levels, involving multiple dimensions including emotions, memories and cognition (Taylor et al., 2010). Better understanding of how to communicate and understand pain is essential to improving outcomes for patients.

### Pain assessment as a social transaction

We draw on the social transaction model of pain communication (Schiavenato and Craig, 2010) to help illuminate key features of SCD and other painful conditions and suggest ways forward. This model assumes that successful pain communication requires mutuality and trust: patients and providers want to minimise pain, and cooperate to transition between pain expression and assessment. Through its treatment of mutuality and trust, the model has potential to offer insight into conditions such as SCD in which trust is at risk. Indeed, the model has already been applied, albeit briefly, to vaso-occlusive crises in SCD (Schiavenato and Alvarez, 2013). On the model, pain assessment comprises the following steps (for a full exposition, see Schiavenato and Craig, 2010). The patient experiences pain, expressing it verbally and non-verbally. This pain is assessed by the provider, referring to its expression, physiological signs, symptoms and clinical information. The provider makes a final judgment, selecting an intervention. Providers differ in how much they consult patients; judgments differ in consensus between provider and patient (Schiavenato and Craig, 2010). We use components from this social transaction model of pain to structure discussion of the literature, beginning from the expression of pain. Each stage raises questions about pain communication for SCD and other painful conditions.^1^

#### (Verbal) expression

Pain expression in general appears ‘context-sensitive and socially organised’ (Heath, 1989, p. 122). Expressions such as cries of pain may be authentic yet tailored to providers’ diagnostic work (Heath, 1989): a patient might express pain the first time a provider manipulates the painful body part, suppressing pain after communicating the diagnostic information (Heath, 1989). Pain expression can also be negotiated: in everyday interactions, children and their parents play an active role in (re)formulating the severity, legitimacy and authenticity of children’s pain experience (Jenkins, 2015). In the case of clinical scales, the role of social and contextual factors in shaping verbal expression plays out in the following tasks for patients:

(1) Interpret the scope of the question (How much pain are you/have you been in?): select the relevant dimension of pain, the relevant time scale, and so on.
(2) Interpret the scale: what do the numbers represent?
(3) Translate the relevant dimension of experience into a number (and any verbal description).
(4) Adjust the rating, if necessary, to achieve one’s goals.

##### Task (1)

Since pain is multidimensional (see the section “Types of scales and their limitations”), questions about pain could in principle refer to any dimension. Provider and patient must coordinate on the intended dimension(s). Some scales probe non-sensory dimensions, such as interference with enjoyment of life or effects on sleep, mood and stress (Giannitrapani et al., 2019). At least in the UK, however, clinical guidelines for SCD refer to the Visual Analogue Scale or equivalents (NICE, 2012), and therefore assume as a standard a unidimensional scale intended to assess the sensory dimension of pain (Hawker et al., 2011).

Even within the sensory experience, pains may have different qualities, which may not be detectable by a scale that assumes a single dimension. These dimensions may be important for pain management. There is limited work on different pain qualities in SCD (Coleman et al., 2016). However, Adegbola et al. (2012) suggest that there are subtle differences in quality between chronic and acute SCD pain. This point may apply to numerous other painful conditions. Relevant, here, are findings on various chronic pain conditions. Patients with osteoarthritis differentiate sensory qualities in soft tissue, the bones, or migraines, and may experience pains in multiple body parts (Dannecker et al., 2018). Similarly, SCD patients may experience pain in multiple body parts, with acute pain overlapping with chronic pain (Taylor et al., 2010).

Temporal aspects of pain add further complexity. When a scale refers to past pain, patients must also identify and aggregate relevant pains, deciding how to report fluctuating pains, recurring or migratory pains (Broderick et al., 2006; Dannecker et al., 2018), which are typical of SCD (Ballas et al., 2012; Taylor et al., 2010). Rheumatology patients describe strategies for fixing scope such as selecting the most painful body part, excluding ‘manageable’ flares or replacing the pain under discussion with more relevant pain (Broderick et al., 2006; Dannecker et al., 2018).

For better pain communication and management, it is crucial to understand how patients experience this process of interpreting questions about their pain, and whether they have strategies to address the challenges of fixing the scope of these questions.

##### Task (2)

Numerical scales require interpretation. While some scales guide interpretation with verbal anchors for numbers, patients must interpret these anchors, relating them to their experience (Giannitrapani et al., 2019). One issue is interpreting endpoints. Patients with various chronic-pain conditions show confusion. Some interpret ‘No pain’ as their usual pain; some interpret the maximum value as their usual, or usual worst, pain, not worst imaginable pain (on chronic limb and neuro-pathic pain, see Robinson-Papp et al., 2015; also on chronic limb pain, see de Williams et al., 2000). Some doubt they can imagine extreme pains (on osteo-arthritis, see Dannecker et al., 2018). Unless told otherwise, patients might understand midpoints as typical pain in some reference population: pain rated 5 is ‘just average’ (Schwarz, 2007). SCD patients report they are aware of the potential for misunderstanding if they give a pain rating as it relates to their own past experience but are understood to have given a rating compared to a less painful past experience, or clinical benchmark (Adegbola et al., 2012). They acknowledge, in other words, uncertainty about the meaning of numbers.

##### Task (3)

Patients must translate experience into numbers. For anyone in pain, it is a complex process to take a pain experience and quantify it. Pain ratings are sensitive to non-sensory factors such as expectations (Brown et al., 2008) or subtle comparisons with other recent pains (see Watkinson et al., 2013). Such factors can mean that a pain rating does not reflect the underlying medical condition (Watkinson et al., 2013). These issues do not appear to have been explored for people with SCD, who may face an additional challenge: that their pain may be so complex that it is especially hard to describe verbally (Ciribassi and Patil, 2016), let alone in numbers. Patients with SCD may rely on analogy and metaphor in describing their pain experiences (Adegbola et al., 2012; Coleman et al., 2016).

##### Task (4)

There are social reasons to adjust ratings away from experience^2^; merely being observed can lower pain ratings (Hadjistavropoulos et al., 2011). When patient and provider are race-concordant, minority patients tend to report worse pain (Hsieh et al., 2011). Concordance is unlikely with conditions like SCD that primarily affect people from Black African and African-Caribbean ethnic minority groups (Hickman et al., 1999) in England, even though healthcare providers are not all white.

Patients may adjust their pain communication strategically, further complicating adequate communication and understanding of pain, and potentially affecting treatment. For example, children with SCD report understating pain to avoid worrying their peers or parents (Atkin and Ahmad, 2001; Marlowe and Chicella, 2002; Miles et al., 2019; Renedo et al., 2020) or to avoid making a fuss at school (Renedo et al., 2020). Adults with SCD report concealing their suffering to avoid social alienation (Umeh et al., 2017). More generally in painful conditions, adults may suppress pain to deny the worsening of a condition (Schiavenato and Craig, 2010); older patients, to avoid changes to living arrangements (Hadjistavropoulos et al., 2011). Patients may overstate pain, for instance to optimise treatment outcomes (Hadjistavropoulos et al., 2011; Schiavenato and Craig, 2010; Tait and Chibnall, 2014) or elicit sympathy (Schiavenato and Craig, 2010). And, as we saw above, it remains to be seen whether patients with SCD replace current pain with a more relevant pain, say, to anticipate future pain and secure appropriate treatment for fluctuating pain.

Patients and providers can differ in all tasks above. In a real life setting, patients and providers must make complex, uncertain inferences about the meaning of ratings – and little is known about how this happens in practice for patients with SCD or about the role of social and contextual factors in expression of pain.

#### Provider assessment and final judgment

The remaining parts of the model focus on actions of, and influences on, providers.

Providers use various information sources in assessing pain. One source is patient self-report, including ratings on a scale; other sources are patient behaviour, signs and symptoms (Schiavenato and Craig, 2010). Providers combine information into a final judgment on the patient’s pain (Schiavenato and Craig, 2010). Since we have already identified interpretative challenge of scales, we turn to non-verbal information, such as facial, behavioural, physiological evidence and background beliefs.

When judging patients’ pain, providers’ assessments may be made relative to recent experience. While experience may lead to skill at recognising signs and symptoms (Schiavenato and Craig, 2010), it may lead to institutional insensitivity and habituation to patients’ pain (Schiavenato and Craig, 2010), perhaps as a result of basic psychological processes (Prkachin et al., 2001, 2004). Patients report that specialists in SCD provide better care and show greater knowledge, sympathy and understanding than emergency providers (Chakravorty et al., 2018; Renedo et al., 2019), suggesting that for SCD, specialists are not inured to extreme pain despite continuous exposure to it.

(Dis)trust and scepticism are also key factors in understanding communication of pain. Negative stereotyping of racial and ethnic minorities may undermine trust and increase scepticism (Becker et al., 2011; Ferguson and Candib, 2002; Staton et al., 2007; Tait and Chibnall, 2014). SCD patients describe their pain reports being doubted, disbelieved and treated as evidence of low pain thresholds or drug-seeking behaviour (Ciribassi and Patil, 2016), observations supported by providers (Ciribassi and Patil, 2016). Some providers – both specialists and non-specialists – directly say that they are sceptical about patients’ pain reports (Labbé et al., 2005; Pack-Mabien et al., 2001; Payne, 2009).

Patients’ coping strategies may conflict with observers’ expectations of how an individual ‘should’ be when they are in pain. For people with SCD, coping strategies can include socialising, watching television or listening to music (Bergman and Diamond, 2013; Ciribassi and Patil, 2016; Marlowe and Chicella, 2002; Pack-Mabien et al., 2001). These patients do not adopt a visible sick role (Ciribassi and Patil, 2016). Alongside reports of extreme pain, requests for strong painkillers and observers’ preconceptions, coping strategies may be misperceived as evidence of deception or being a ‘difficult patient’ (Bergman and Diamond, 2013).

When providers make their final judgment about the pain of the patient, they have multiple sources of information: for instance, patient self-report, patient behaviour, the provider’s judgment, medical records, case histories and medical tests. They presumably select from or aggregate such information (Schiavenato and Craig, 2010). Indeed, clinical guidelines may require providers to combine their observations with patients’ self-report (Johannessen, 2019). Perhaps implicitly, providers may judge the reliability of each evidence source, informed by factors such as those considered in this section. Is the patient trustworthy? Are the records comprehensive? Is the provider confident in the history? Are the tests reliable? Evidence sources are typically partially reliable: human sources (patients or providers) can be confused or mistaken, can mislead or lie; medical tests are imperfect. Sources need not be treated equally: ethnographic data suggest that providers can view ‘objective’ data as less ‘tainted’ or less unreliable (Johannessen, 2019). Sources may also disagree: 50% of SCD patients lack physiological signs during a pain crisis (Jacob, 2001), and if there is no evidence from an expected source – say, expected behaviour is absent – this can be taken to suggest pain is absent or reported pain is exaggerated (Johannessen, 2019). Empirical data are lacking on how providers aggregate evidence in practice.

Provider judgment and final assessment may also contribute to an important feedback loop that is part of Schiavenato and Craig’s (2010) model. According to Schiavenato and Craig (2010), if patients present with pain and are treated with distrust or scepticism, patients may modify their behaviours as a result. This modification may in turn create scepticism in providers, if providers do not perceive the behaviours as sincere (Schiavenato and Craig, 2010).

#### Intervention

Having assessed the patient’s pain, providers then select an intervention, presumably balancing criteria such as the effectiveness of treatment, side effects and concerns about addiction or controlled drugs being traded illegally. The relative weight given to these criteria might change because of (dis)trust. For instance, if providers believe drug addiction is common among people with SCD, they may be sceptical about prescribing opioids (Ciribassi and Patil, 2016). As with provider assessment and final judgment, a key question here is the extent to which patients are aware of such factors and how awareness affects their communication of pain and their experience of pain management.

As evident from the literature reviewed above, pain communication is highly complex. Yet there is little empirical data on how this might affect care. In this article, we investigate some of the key areas identified in the model, using empirical data from interviews with young people living with SCD to illuminate the ways the different theoretical areas of the model might manifest in practice. We examine what this can tell us about how we might improve communication and understanding of pain in clinical settings.

## Methods

We used a longitudinal qualitative design to examine young people’s experiences of living with SCD. Pain management is a key concern for young people (Miles et al., 2019; Mulchan et al., 2016; Renedo et al., 2020). We selected participants for repeated interviews on the basis of their age to capture their experiences in real time during the transition to adult care. We conducted 80 interviews across England with 48 young people with SCD (aged 13–21): 27 one-off interviews (17 with 19–21 year-olds, and 10 with 13–18 year-olds) and 53 repeated interviews with 21 13–18 year-olds, interviewing them 2–3 times over approximately 18 months. Interviews lasted 60–90 minutes, were audio-recorded and transcribed verbatim. Interview topic guides were developed with a young adult with SCD (patient representative in the project) and examined participants’ experiences of receiving healthcare and living with SCD. In the interviews we explored healthcare and social aspects of transitions to adulthood, including experiences of living with pain. Repeated interviews allowed us to capture the unpredictability of pain episodes and ask specific follow-up questions for each individual to revisit issues discussed in the previous interview. The second author conducted interviews at a location participants chose, usually in their homes but sometimes in hospitals. Only the interviewer and participant were present during the interview.

We analysed interviews using an inductive, iterative approach, combining some of the practical steps of Grounded Theory (Charmaz, 2006) and thematic analysis (Attride-Stirling, 2001). The coding frame was developed inductively from the data set and was also based on our a priori interest in understanding young people’s illness experiences in the context of their whole lives beyond the clinical setting. The coding frame was refined alongside data collection and analysis, including via reflective analytical sessions with user representatives in the project. During analysis we took into consideration how the interviewer being a white, adult researcher without SCD influenced interview dynamics with the Black, young, interviewees with SCD. Analytical categories were developed and refined through repeated rounds of coding and ‘memo-writing’ (Charmaz, 2006; see p. 72 about codes and emerging analytical themes), and via the reflective analytical sessions with user representatives (Miles et al., 2018). We examine how young people account for their everyday pain experiences and their experiences of communicating pain in unplanned non-specialist healthcare (Accident and Emergency and when admitted into a hospital general ward).

The study was approved by the London School of Hygiene and Tropical Medicine (Ref 10107) and NHS research ethics committees (REC 15/LO/1135). Participants (16–21 year-olds) and parents/carers of 13–15 year-olds gave informed consent to participate, and younger participants (13–15 year-olds) additionally gave their informed assent. We provided participants with information on referral agencies should they need help with issues raised in the interviews. Quotations from interviews are labelled only with age range to protect anonymity. Each participant received a shopping gift voucher to compensate them for their time.

## Findings

We identified the following themes: (1) the complexities of scale use; (2) the role of provider judgment and final assessment; (3) (dis)trust; and (4) relationality of pain to patients’significant others and to patients’ history. Here we present the findings from these themes, linking them to the themes from the social transaction model.

### Theme 1: The complexities of scale use

#### Interpreting the scope

Patients may feel that their current pain (under treatment and discussion with staff) is not the most relevant to report on. Painful episodes can last for hours, several days or even weeks. Interviewees referenced expectations of how the pain being assessed and treated could rapidly escalate or fluctuate. Interviewee I10 raised the question of the scope of the pain scale directly, distinguishing between current and more relevant future pain, and anticipating future care:

*I had a lot of, er, painkillers that time, I had strong painkillers, erm, so there were a lot of nurses [. . .]* ^3^ *they just kept on coming in and telling me that er, erm, erm, that it’s OK for me to go home ‘cause, ‘cause when they asked me “What’s the scale of, like, one to ten of your pain?”, I told them “at the moment it’s not, it’s not bad, it’s about, er, three or two, so it’s not bad at all”, but I told them that, erm, if I go home, erm, like at that, at that moment if I went home, I would pretty much be back tomorrow morning ‘cause the, erm, medicines that they gave me would wear off. But I don’t think they were listening to me [. . .] they must have thought that maybe I didn't know what I was talking about. [I10 16–18 years old]*

Interviewee I1 (below) told us how her interpretations of her pain differed from staff assessments. For instance, her particular sensory experiences indicated to her that the acute painful episode under analgesic treatment was unresolved and current pain was likely to change. Staff dismissed her assessment.

*[staff at A&E] they were like, “Oh, um, like I think you can go now, you’re better with this”. But then I wasn’t feeling better at that time, because I just came in. And then he was like, “Oh, um, we’ll give you more medication [analgesia], and then after we see this”. And he was proper, he examining me, and he was proper like watching me. But then I knew how I felt inside. But then he was telling me how I was all right [. . .]. I was still ill [. . .]I felt annoyed [. . .] because, like, he doesn’t know how I, I was feeling at that time, but then he was telling me that I was fine.[. . .]I do tell them that I’m not ready [to be discharged], but then they feel, they feel like I’m ready. [I: What makes you feel you are not ready?] I’ll sometimes, like, a part of my leg could just start aching or my back, and then I just feel like I’m not ready, but then if I feel like I’m able to walk and all of that, then I feel like I’m ready. [I1 13–15 years old]*

Some interviewees drew on their sensory experiences to estimate how long their acute painful episode would be, and said that they could tell when current doses of analgesia would not be enough and further doses would be needed until the painful episode subsided (Z1 below).

*If it’s tingling, that’s when I know it’s going to be just a few days of a crisis [acute painful episode]. But if it’s pins and needles then I know that it’s going to be much longer than a few days [Z1 19–21 years old].*

Interviewees talked about the sequence of pain events in an acute painful episode, explaining how pain differed in quality and degree at different stages: more or less manageable; more or less diffused. During a painful episode involving a sequence of many pains, some specific pains were more difficult to locate, happening in multiple areas simultaneously (I3 below). One participant (E5) explained how because the pain is in the blood vessels, which run ‘everywhere’, it was felt deep in the muscles.

*Stage three [of the painful episode] is when the sharpness overtakes the flames and, erm, you can locate that pain area. But in stage two you can’t because it’s still developing, and stage one it’s like you are waiting for, erm, because stage one it could be anywhere, so you don’t know precisely where the main point is. So, I have to wait until that’s done and then stage two: you can feel it developing but in small areas so you have to, it’s hard to look, pinpoint, exactly the location; and then stage three is the main location of where it’s fully developed and you feel the arrow head and then the flames. [I3 16–18 years old]*

Interviewees’ narratives indicated that participants were discounting manageable pains: whenever self-management was possible, young people with SCD would conceal signs of being in pain, trying to carry on with life.

*Sometimes I think the medication’s [analgesia] unnecessary so I can take the pain and then it will wear off. Sometimes, maybe at school I can, I’ll have back pain, and I won’t take medication and I’ll just sit there. Like say I’m with my friends – I think this happened three months ago – I had [long pause: 2s] back pain in my back. I could sense it was coming, and then it was really hurting and I had to go to class. And it was really bad but somehow [long pause: 2s] it lasted for about three hours and then it (.)* ^4^ *subsided, (.) and then I won’t take medication [. . .]. Maybe it’s because I have a high pain threshold, but only when the pain gets really bad (.) I’ll take the medication. [E1 19-21 years old]*

#### Translating pain into numbers

Interviewees cited differences between SCD pain and other pain [I6] and difficulty in translating SCD pain into numbers:

*[In emergency care services they are] slower with giving the medication, the pain relief, [. . .] they just like think you’re a drug addict or something, [laughs] he just wants to feel (.) some morphine. [. . .] Because they’re always like, [. . .] “how hard” – like: “on a scale of one to ten how tough’s the pain?” And if you say anything below, like, five, then they’ll say: “we’re not going to give you no morphine [laughs] because you don’t need it”. But really you do, even when it’s at, even when the pain’s at, like, a five level, you need the [morphine]. [I6 19–21 years old]*

Interviewees showed evidence of finding their pain ineffable. Given the option to use spoken words or drawing, young people with SCD struggled to explain their pain experiences. They expected that others without SCD would not understand the type and degree of pain they experienced, not having experienced the same type of pain. They said that this made describing pain harder. During interviews, participants used tactics such as describing their pain as something inflicted by others, like being stabbed, shot or drowned. They used imagery to represent pain, such as knives, arrows, an ever-present raincloud or a boomerang that kept hitting them.

People with SCD told us informally how their experiences, such as not being given pain relief when they gave a particular pain rating, taught them to score their pain to guarantee a particular type and dosage of analgesia. Their history of pain and treatment influenced their pain expression, and they had learned about providers' role in interpreting the severity and authenticity of their pain expressions.

### Theme 2: Provider assessment and judgment

Our interviewees mention providers comparing them with other people with SCD or other conditions [O1]. They emphasised the idiosyncrasy of SCD pain, explaining how each person is affected differently. Their healthcare experiences during transition to adulthood had made them aware of the limited knowledge of SCD amongst non-specialist hospital staff and the problems this could cause for their pain to be recognised and treated appropriately.

*Nurses asked me, she said, “Oh, you have sickle cell”, I said “yeah”. She said: “how long have you had it?” And I was just like, “I was born with it” [laughter] kind of thing. So I just thought they don’t understand really. [. . .] The last thing I wanna do when I’m sick [in pain] is start explaining stuff all the time to each person that comes. [U9, 19–21 years old]*

*They’ll [nurses] assume that. . . let’s say my pain score is at a five or a four, when really it could be at an extreme ten and still be there laughing and being happy. [. . .] Sometimes [doctors] they’re not trained or the, their speciality of, maybe they might be more into cancer research or to people with different, different, different cases than sickle cell. Sometimes they’ll think that, they’ll try and compare, I feel sometimes they try to compare the two, like two different cases with one another and sometimes they get it twisted because it might not be the same case. Maybe that person might be feeling pain, but they need to understand that my pain might be ten times worse and that I could be, other people have a different pain tolerance. Some people can be crawling, or crawling around because they’re in pain or scream, sometimes people are even cursing, but with me I’m usually with pain I’ll try to be calm and breathe in and breathe out, so that it’s more calming and try and get control of the pain. [O1 13–15 years old]*

Interviewee O1 explained that if the nurses saw her laughing, they interpreted her pain score as a five or three, when she would have rated it a nine. Our interviewees said the fact that their pain was invisible made it difficult for them to have the pain acknowledged by healthcare staff:

*Of course now [pain] it’s not visible at all ‘cause I could just hold it in for as long as I can. But I think maybe a few years ago I, it is visible. [. . .] If you can’t see that that person’s in pain and they are then, er, it’s gonna make it difficult to get them treated because, you know, he looks fine. [. . .]It’s like in, in A&E when I was in the emergency, if you look fine or you look like you’re not in pain then they’re gonna take a very long time to, you know, get in contact with you and get you sorted out. So I think it’s, it was much easier when I was a bit younger to get seen because you’d be screaming, you’d be in a lot of pain and they could see it. And, ‘cause they can see it, they can react to it. But yeah, now, now, now it’s just, er, it’s not as easy to, to get across that you’re in a lot of pain ‘cause they can’t see it. So yeah, it is a bit more difficult, visibility. [O4 19-21 years old]*

Our interviewees emphasised how problematic it could be to be in pain but not show it – reporting being in pain was not enough. Interviewee U9 explained that he had been having painful episodes since he was very young, and so had become good at coping with it. For this reason, he did not ‘look as sick as other people’ when he was in pain, which ‘work[ed] against’ him. He said this translated into being discharged too early or not being admitted to hospital at all:

*Sometimes, they [healthcare staff on the ward] don’t really listen to how you feel at times, they kind of just look at you and think, “Oh, you look OK, we’ll let you go”, sort of thing. And even sometimes like I’ve had pains in my legs and I’ve been to [hospital] and, erm, they, they, they’ve like just, erm, not admitted me when maybe they should have. [U9 19-21 years old]*

### Theme 3: (Dis)trust

Some interviewees said they knew that non-specialist providers might limit their access to the morphine they needed:

*There was one time where I needed Oramorph and you know the whole situation where there are some, some patients who get addicted to it. [. . .] So they, they might think that I was gonna get addicted to morphine or they [doctors/nurses on the hospital ward] might think that I was giving them the wrong, wrong instructions, that I’m not supposed to have morphine. So then it makes it a bit, a bit more difficult to communicate. [. . .] I remember asking for morphine and they just, they didn’t want to give it to me. They, they thought I wasn’t in the right place to ask for it or something like that. [. . .] They were just like, um, “You know, we’re gonna have to ask the doctor”. [O4 19–21 years old]*

Interviewees told us that they experienced delays and this made them distrust non-specialist providers. They gave this as a major reason for avoiding hospital during a painful episode. Z2 told us she had been ‘ignored’ when she asked for pain relief, which made things worse for her and changed her relationship with hospital staff:

*Once that had happened I sort of changed how I behaved in hospitals. Like I tried to make sure that I was heard, because I didn’t want to fall back into that time. [. . .] Even now when I go back I still don’t fully trust them, even in different hospitals, it, it doesn’t really matter to me. I try to avoid the hospital as much as I can, not specifically because of that experience, but that has had a very big impact on my trust with, like, doctors and nurses, in understanding sickle cell. [Z2 19–21 years old]*

### Theme 4: Relationality to others and to personal history

Participants accounted for their pain through and against others. The others’ gaze was explicit in their narratives. Participants introduced parents and significant others into their accounts, often seeming to prioritise how pain affected others. Participant Y5 introduces her mother’s experience, seemingly privileging her mother’s suffering within the narrative:

*I used to have literally a [pain] crisis nearly every day, so there was some times my mum couldn’t bear it, every week I’d be in hospital for about a weekend or something like that. [Y5 16–18 years old]*

Similarly, I3 prioritises how her mother is affected by her pain, concealing her pain to protect her mother and to protect herself from seeing her mother suffer:

*When I get a crisis [. . .] my mum, like, worries about me and then I worry, like, that kind of breaks my heart. [. . .] Sometimes I don’t show it but I do actually worry about my mum. It breaks my heart when I see my mum, like, upset when I’m, when I’m in pain and stuff like that because I don’t want her to worry about me like that. [I3 16–18 years old]*

Participants brought others into their accounts when describing their pain episodes as being observed or ‘on stage’ (A5): subject to others’ reactions or judgements. A5 also told us about the unpredictability of painful episodes and her worries that she might ‘scare [friends] off’ if an episode started when she was with them:

*I wouldn’t, I wouldn’t draw myself alone [A5 on how she might make a drawing to depict her pain], um, I, I think I might actually, like, draw myself on stage in pain and, um, ‘cause, ‘cause, when I do have a crisis, my, my parents, you know, they, like, tell the whole family what’s happening and, um, so there are a lot of people involved. [A5 16–18 years old]*

Participants mentioned not wanting peers or family to see them in pain to avoid shocking or worrying them, or to avoid being judged as overreacting. Participants talked about suppressing pain expression in various contexts, concealing pain or ‘control[ling] [pain] in other people’s eyes’ [Z2; 19–20 years old] and learning from childhood how to ‘mask’ pain – to ‘put a brave face on’ [Z1; 16–18 years old]. U8 talks about the social dimension of pain more explicitly, saying how significant others are affected by it, indicating how pain is made sense of through social relationships.

*It’s normally not just, um, it, obviously it’s not just the person who’s having the pain, it’s the people around them that have to do other things for them, so then they have the sort of stress, as well. [. . .] Sometimes it feels like you’re annoying other people just, like, by having pain and, like, stopping what they’re [parents] doing for, for yourself, um, so then it just, like, you don’t want them to feel that they have to stop everything and that they can, um, do things for themselves. [U8 13–15 years old]*

*I feel a bit like a burden to my parents ‘cause I have pain all, all the time and they have to deal with it all the time. And, um, part of me feels like I’m doing them a favour by staying in my room so that they can just get on with whatever they have to do and not worry about me. [A5 16–18 years old]*

Participants described how their experiences changed over time and how they learned to be resilient:

*You develop, um, what’s called, pain, what’s it called? Um, where you are more tolerant to pain. [. . .] So, no, when it’s worse because if it’s worse I can’t move but say, like, it’s medium or low then I will try and go home, that’s if I was out. [I3 16–18 years old]*

## Discussion

Pain scales may help provide thresholds for pain-management decisions, and perhaps create an appearance of objectivity in pain communication. Yet our findings show there are important social and contextual influences on how patients express pain and how providers interpret it. These influences will not be detected by studies that validate scales under experimental conditions. This is likely to apply to other conditions where pain is complex or lacks overt physical signs.

The question of how patients reach pain ratings is important. Our interviewees identified difficulties with scales. They indicated difficulties with scope, pointing to sequences of pain qualities that allowed them to predict how their painful episodes would evolve, qualities not detectable by scales or appreciated by providers. As with the rheumatology patients discussed in the introduction, our participants said they discounted some pains when making their judgments, and anticipated future pains. They were concerned, too, about the meanings of the numbers and used analogy and metaphor to communicate their pain to us. They also recognised the importance of expertise, citing non-specialist providers’ lack of expertise as a reason for poor experiences.

Our findings also show the importance of distrust. Interviewees were aware of providers’ likely scepticism and scrutiny of their behaviour, as well as providers’ conflicting goals and limited knowledge of SCD. After patients learn that staff do not always understand their pain, resorting to strategic uses of scales may be their best option to secure appropriate treatment. Interviewees were also aware that their coping behaviours might be misperceived by providers, adding another complication. These points align with the first stages in the distrust feedback loop proposed in the social transaction model; to study this feedback loop further, it will be important to explore how providers respond to interviewees’ strategies.

The social transaction model helped to illuminate pain-scale use. But our interviews suggest important limitations. The model considers patient and provider, but does not make it explicit that the social dimensions of pain go beyond these two-way interactions, to include relationships with others. Our interviewees often introduced significant others into their narratives, prioritising others’ feelings and experiences of their painful episodes. Our findings suggest that pain communication is shaped by relationships with multiple others, whether real (e.g. carer present during the painful episode) or imagined – for instance, a memory of people encountered and their reactions to painful episodes and pain reports. While the model acknowledges external influences on the ‘patient-clinician dyad’ (Schiavenato and Craig, 2010, p. 671), our findings suggest there should be greater emphasis on a more nuanced understanding of relationality in pain communication and experience.

Another limitation lies in the assumption that provider and patient both wish to minimise a particular pain. While this assumption of mutuality may ultimately hold, a patient may desire a particular type and dosage of analgesia, while the provider may want to limit access, fearing dependency, addiction, or illegal trade in controlled drugs (Aisiku et al., 2009; Ciribassi and Patil, 2016; Jacob, 2001; Payne, 2009). Our interviews show that patients are aware of these complications, raising questions about the assumption of mutuality in SCD care.

The social transaction model emphasises the damaging effects of distrust and scrutiny (Schiavenato and Craig, 2010), but leaves unaddressed the basic mechanisms through which the effects arise. We have shown how important distrust may be for people with SCD, underlining the importance of studying the precise effects of distrust on the assessment process for pain associated with any condition. (Dis)trust may also determine how providers aggregate information, an underexplored process.

Our findings broadly support previous work that suggests pain scales for SCD are not adequate (see Adegbola et al., 2012; Coleman et al., 2016). But how might pain assessment be improved? One option is to assess pain more qualitatively whilst bearing in mind that speaking while in pain can be burdensome for patients. As we have seen, patients with SCD distinguish different qualities in their pains and use analogy and metaphor to describe pain. Qualitative descriptors also need interpretation, and do not avoid issues with trust. But if commonalities could be found in patients’ descriptions – and existing work is promising in this regard (Dampier et al., 2002; Franck et al., 2002) – then these commonalities could form the basis of richer assessment tools. There is considerable scope here to explore the effectiveness of these descriptors in predicting and distinguishing pains through experience-sampling studies.

Lack of trust and compassion for patients was often a problem for our interviewees whose reports of pain were disbelieved and whose pain medication was withheld or delayed by the non-specialist healthcare staff they were obliged to rely on in emergency care. To improve pain management and provide care that recognises the expertise of patients in their own bodies, we recommend providers be trained in communication skills and compassionate skills, to elicit and respond to patients’ voices, and involve them in shaping the care they receive (see also Miles et al., 2019; Renedo et al., 2019).

Future research might improve our understanding of pain communication in contexts such as those in SCD. The literature and our interviews reveal uncertainty about meaning – how questions are interpreted and experienced is translated into numbers. Some patients rely on metaphor and imagery rather than quantification to describe their pain. The question arises of how providers and patients navigate this uncertainty about meaning in SCD, suggesting a need for further studies in specialist clinics or simulations (with SCD patients) of emergency care where provider-patient interactions can be observed. Observations of pain interactions in other contexts have revealed subtle social influences on pain communication, such as how the expression of genuine pain can be tailored to providers’ diagnostic work (Heath, 1989) and how children's pain reports can be reshaped during interaction with parents (Jenkins, 2015). Similar studies on SCD could refine our understanding of how pain is communicated between provider, patient, and significant others. It would also be useful to interview providers about how they use scales: how they interpret extreme ratings, whether they rescale ratings, whether they consider non-sensory aspects, and how they combine evidence into a final judgment.

Intervention studies could also help illuminate this area further: for example, experiments testing the effects of participatory dialogues between provider and patient to explore the parameters set out in this article, and facilitate a social process of mutual learning about the goals of both provider and patient. Such participatory interventions could help to raise providers’ and patients’ critical awareness (Freire, 1973, 1990) of how the relationality of pain shapes communication and support them in collectively devising a plan for improving pain assessment in clinical contexts.

## Summary

While very commonly used, pain scales have numerous drawbacks and are prone to profound social and communicative influences that may not be adequately taken into account. We identified key factors: the meaning of the scale; relationship to social, statistical and psychological factors; and trust. There is considerable uncertainty inherent in scale use, and considerable scope for future work to explore this uncertainty and improve pain communication.

## Notes

1. These stages are only part of Schiavenato and Craig’s model (Schiavenato and Craig, 2010), which includes various contributing factors and intervening steps. For present purposes, the stages are most useful. We do not strictly distinguish between the stages and other parts of the model. See (Schiavenato and Craig, 2010) for more detail.

2. See, also, Schiavenato and Craig's step ‘Display’.

3. Omission markers, *[. . .]*, indicate where we have cut text from the transcript.

4. (.) indicates a brief pause.
